# Age-related changes in somatic condition and reproduction in the Eurasian beaver: Resource history influences onset of reproductive senescence

**DOI:** 10.1371/journal.pone.0187484

**Published:** 2017-12-05

**Authors:** Ruairidh D. Campbell, Frank Rosell, Chris Newman, David W. Macdonald

**Affiliations:** 1 University College of Southeast Norway, Department of Natural Sciences and Environmental Health, Bø i Telemark, Norway; 2 Wildlife Conservation Research Unit, The Recanati-Kaplan Centre, Zoology Department, University of Oxford, Tubney, United Kingdom; Leibniz Institute on aging - Fritz Lipmann Institute (FLI), GERMANY

## Abstract

Using 15 years of data from a stable population of wild Eurasian beavers (*Castor fiber*), we examine how annual and lifetime access to food resources affect individual age-related changes in reproduction and somatic condition. We found an age-related decline in annual maternal reproductive output, after a peak at age 5–6. Rainfall, an established negative proxy of annual resource availability for beavers, was consistently associated with lower reproductive output for females of all ages. In contrast, breeding territory quality, as a measure of local resource history over reproductive lifetimes, caused differences in individual patterns of reproductive senescence; animals from lower quality territories senesced when younger. Litter size was unrelated to maternal age, although adult body weight increased with age. In terms of resource effects, in poorer years but not in better years, older mothers produced larger offspring than did younger mothers, giving support to the constraint theory. Overall, our findings exemplify state-dependent life-history strategies, supporting an effect of resources on reproductive senescence, where cumulative differences in resource access, and not just reproductive strategy, mediate long-term reproductive trade-offs, consistent with the disposable soma and reproductive restraint theories. We propose that flexible life-history schedules could play a role in the dynamics of populations exhibiting reproductive skew, with earlier breeding opportunities leading to an earlier senescence schedule through resource dependent mechanisms.

## Introduction

Senescent declines in reproductive success, somatic condition and cohort survival rate [1,2] influence the schedule of lifetime breeding success [3–7] in mammal species. A variety of theories have been conceived to explain the mechanism of senescence [8–14], many of which are based on the observation that because the cumulative risk of extrinsic mortality increases over time, the force of selection reduces with increasing age [9,10] as future reproductive value declines. In addition, natural selection is hypothesised to mould senescence synchronously across traits [9], but see [7]. In this study, we examine the effects of resource availability on senescence in reproductive and somatic traits in relation to four theories that have been proposed to explain the prevalence of senescence, or commonly observed changes in reproduction, somatic condition and survival with age: the disposable soma theory [11–13]; the reproductive restraint theory [13]; the constraints theory [15–17]; and the selection theory [16].

In wild-living populations, subject to natural selection, the annual and life-time availability of environmental resources can ‘stress’ organisms [18], either directly through actual insufficient availability, or indirectly through physiological constraints on an organism’s capacity to acquire them sufficiently [19–21]. This leads to trade-offs in energy allocation, especially when food supply is limited, with a division between reproduction and investment in somatic maintenance and repair, to ensure continued survival (or ‘longevity assurance mechanisms’, [22]). Theories linked to resource-dependency include the ‘disposable soma’ theory ‘DS’ [11–13] and ‘reproductive restraint’ theory ‘RR’ [13]. DS posits that the trade-off between energy allocated to reproduction and investment in somatic maintenance and repair leads to deterioration in the organism’s body with age [11]. RR posits that when extrinsic mortality risk relative to intrinsic mortality are low later in life reduced reproductive effort may arise in order to enhance survival probability and provide additional future breeding opportunities. In wild populations it can be difficult to disentangle the trade-off involved in these theories from genetically mediated age-related effects. Both DS and RR predict declines in female reproductive output with increasing age, while DS also predicts a decline in somatic (body) condition. To the extent that any trade-off occurs, individuals consistently exposed to poorer resource availability over their reproductive lifespan (their resource history)–for example because they live in habitat that is of lower quality than their conspecifics–will exhibit a concomitant reduction in investment in at least one of reproduction or somatic repair and maintenance. Therefore, an influence of individual resource histories on senescence is predicted from both DS and RR theories.

Conversely, lower reproductive success in early life may be explained by the constraint theory (‘CT’, [15–17], which predicts that poor physiological condition, or inexperience, can limit breeding in younger individuals [16]). Thus CT predicts lower early-life reproductive output and somatic condition.

Potentially pertinent too is that, at the population level, less fit individuals such as those less able to acquire resources are likely to die sooner, causing selection for superior phenotypes to inflate per capita reproductive rate in the surviving population; termed the selection theory (‘ST’, [16]). A cohort would thus become comprised by a greater proportion of fitter individuals over time [23–25]. Like CT, ST predicts higher late-life reproductive output and somatic condition. Unlike CT however, these patterns will only be detectable at the population level.

To investigate these potential resource-dependent effects on reproductive and somatic senescence we analysed 15 years of data from a wild Eurasian beaver (*Castor fiber*; henceforth ‘beaver’) population, collected in Telemark, Norway. The beaver provides an informative model species for examining senescence, being long-lived (up to 20 years in the wild; noting that in our study population, mean age at death for dominant adults was *c*. 7 years [26]). Beavers are also highly territorial, where territory borders can remain stable for several years [27], and exhibit changes in breeding success with age [28]. In terms of dietary resources, beavers are large (> 20 kg), herbivorous, semi-aquatic rodents [29,30] that forage predominantly on deciduous trees, consuming leaves, twigs and bark [31,27]. Pertinent here is that previous research on this same study population has found that higher rainfall correlated with lower resource availability, due to trees near water level producing poorer forage in wetter years, due to water logging [31]. This resulted in lower body weights for adults, yearlings and offspring (kits), fewer kits weaned per breeding female, and lower weaned kit and yearling survival rates [26,31].

In terms of socio-spatial distribution and reproductive patterns, beavers are behaviourally monogamous, tending to remain faithful until a mate is displaced or dies [30,32] (but see [33]). Only the dominant pair in a family group breeds [27,29], creating reproductive skew (*sensu* [34]). Extended family groups can develop due to the retention of philopatric offspring [27,35], although during the study period groups rarely (<10% of occasions) exceeded five individuals (mean group size in this population = 3.42 ± 1.73 SD), although we recorded 11 individuals in one territory in one year. Allo-parenting thus has the potential to play some role in reproductive success, or offspring quality. Litters of 1–5 kits (although litters of 4 or more are unusual in the wild) are born around mid-May in Norway [27,36] and emerge from the natal den or lodge when weaned, one to two months later [29]. Sexual maturity is attained at age 1.5–2.5 [29], whereupon offspring may disperse to fill any available territory vacancies [27,37].

Here we examine changes in adult body condition in both sexes along with changes in reproductive success with age in females (breeding frequency and offspring number and quality). We also examine to what extent long-term resource (food) availability, mediated by territory quality, influences reproductive senescence relative to short-term resource availability, mediated by rainfall.

## Materials and methods

### Study area

The study site was centred on three rivers, the Straumen (N59.30, E9.09 to N59.91, E9.19), the Gvarv (N59.41, E 9.12 to N59.37, E9.20) and the Sauar (N59.47, E9.31 to N59.37, E9.25), in southern Norway, with one beaver family group per 2km of river length [27]. Riparian woodland bordered these rivers, even along agricultural stretches, dominated by grey alder (*Alnus incana*) and, to a lesser extent, willow (*Salix* sp.) and bird cherry (*Prunus padus*). The beaver population in the study area is open to immigration from similar contiguous riparian habitat. There is no reason to believe that the beavers outside the study area would be any different in terms of life-history strategy. Beavers have been in this area since 1920s and there were no substantial changes in population density over the study period [38]. For further details on the study area, see [31].

### Beaver trapping and sampling

Between March and November from 1998 to 2012, beavers were live-trapped from a motor-boat using hand-nets [39]. Mean captures per year per individual varied from 1.7 to less than one. Captured beavers were restrained in cloth sacks, sexed by the colour of the anal gland secretion [40], weighed (to the nearest 200 g), and body length (cm) was measured along the spine from nose-tip to base of tail. Beavers were tagged with a microchip (Avid or Trovan) and marked with colour-plastic (Dalton) and metal (National Band and Tag Co.) ear-tag combinations. An animal was assumed to be resident if it was trapped or sighted in the same territory more than once >24h apart, or was seen interacting non-agonistically with other known residents [26]. We defined trapping effort per territory as the number of nights spent trapping along each river section (Straumen, Gvarv, upper Sauar and lower Sauar) in year *n* and *n+*1.

Age-class was assigned on first capture, based either on weight [38] (see also [30]) or previous trapping history (year 0 = kit, year 1 = yearling, year 2 = sub-adult and ≥ year 3 = adult, where ‘year’ ended on 31^st^ Dec). Animals first trapped as kits or yearlings, and those sub-adults that could be aged with confidence, were assigned their actual age–referred to here as ‘known-age’ individuals [38]. Many of the individuals measured were adult when first trapped (e.g. 78% of individuals in the model of somatic senescence and 82% of dominant females in models of reproductive senescence), either because they were already adult at the beginning of the study, they evaded detection over their first three years, or they were recruited from outside the study area. Animals first trapped as adults were assumed to be three years of age (minimum age) and sub-adults two years of age. Although including minimum age in addition to known-age individuals made it possible that the age at which senescence commenced could be underestimated, (but not over-estimated), this allowed us to include a larger sample of females. The probability of detecting senescence was therefore more stringent, permitting us to identify relationship minima with confidence; that is, any signal for senescence had to be stronger to be apparent. Only one dominant female occupies each territory, identifiable from greater body weight than other same sex group members and signs of lactation (nipple length > 0.5cm). These were assigned maternal status, based on their trapping and sighting history. Furthermore, females dispersing into a territory were assigned the dominant breeding position (generally corroborated by the disappearance of the previous incumbent). Without contrary evidence, we assumed individuals maintained their dominant status until they disappeared or died [26]. Since dominant adult beavers in the study area very rarely move out of their territory to breed elsewhere (see below), we assumed that disappearances arose from death of the individual. Of the 39 breeding females used here, 13 were still alive at the end of the study. Fifteen of the females held their dominant adult breeding positions at the start of observations on their territory, of which three were removed from the study area as part of an international reintroduction programme, 11 disappeared or died during the study, and one was alive at the end of the study, having been under observation for 14 years. Thus we observed the entire reproductive lifespan for the remaining 12 (31%) of the females, only the latter part of the reproductive lifespan for 11 (28%) and only the early part of the reproductive lifespan for 12 (31%).

Metrics of reproductive success were derived from the number of kits trapped per year plus the number of yearlings trapped the following year that were not trapped as kits previously (mean first year mortality 8%, [26]). Two-year olds (mean mortality age 1–2 28%, [26]) and unmarked offspring clearly resident in the territory were also included in reproduction metrics. Over the whole study, these comprised respectively 48%, 25%, 17% and 10% of all observed offspring. Offspring body metrics were taken from the first records of individuals per year, controlling for season to account for on-going growth. Sample sizes are provided along with each model, below.

### Territory quality

Territory borders show little change between years and dominant adult beavers rarely change territory [27]; therefore, territory quality provided a consistent proxy of resource history for each reproductive female. Territory quality was defined as the total availability of deciduous saplings (principal food source) within each territory (S1 File). Based on this metric, we divided territories into four quality categories (*TQ*_*4*_): 1 (lowest quality) to 4 (highest quality). Since territory borders typically remain stable over time, only four of 39 females changed *TQ*_*4*_ over the study period, where one change was because the female moved territory and the other changes resulted from shifts in territory borders [27].

### Rainfall variables

Higher rainfall has been established to correlate with lower resource availability to beavers, due to trees near water level being prone to water logging and thus producing poorer forage in wetter years [29]; therefore, we used rainfall as a proxy of annual variation in resources. Daily rainfall data, 1997 to 2012, were sourced from the Lifjell weather station (354m a.s.l., 59°455 N, 09°037 E) using *Eklima* (*http*:*//eklima*.*met*.*no/*), which correlated well with rainfall measured on the study rivers [31]. To account for exceptionally high rainfall in July 2007 [31], we adjusted for spring-summer (Apr–Sept) run-off; this was unnecessary for autumn rainfall (Aug–Oct).

### Theory and calculation

Studying wild aquatic rodents, able to retreat into lodges, is challenging. Therefore, studies of senescence on wild-living animals have largely been limited to more tractable ungulate species. Consequently analytical procedures had to be adapted to best utilise available data.

For analyses of annual reproduction, we excluded seven territories with less than four years of data. Due to stable monogamy, breeding males and females were the same (minimum) age in 75 of 132 (57%) instances, with only 11 (8%) instances, 1998–2008, where an adult of either sex ≥8 years was paired with a mate of ≤5 years. Due to the sexes aging in parallel, we focused on the influence of female age on reproductive success, whereas for analyses of adult body weight, we were able to examine the influence of age for both sexes. Relevant sample sizes are provided along with each model, below.

All statistical analyses were conducted in the *R* environment [41]. Unless otherwise stated, all linear mixed models (LMMs) and generalized linear mixed models (GLMMs) were run using function *lmer* in the *R* package *lme4* [42]. From each global model, we specified a subset of candidate models that included all possible combinations of fixed effects, retaining only essential variables in all candidate models (S1 File). Akaike Information Criterion, controlling for small sample sizes (AICc) was used to weight models within candidate sets in the *R* package *MuMIn* (v. 1.7.11, see [43]). We used Akaike weight based averaging, over all models within AICc = 4 of the single most supported (lowest AICc) model (the top model set), to calculate parameter estimates and associated 95% confidence intervals (CIs) [44], where the exclusion of zero inferred significance. Predicted values, with 95% prediction intervals calculated from the most supported models, were then used to explore significant interactions. Little or no overlap of prediction intervals inferred a significant effect. Global models were assessed for over-dispersion (Gaussian models) and homogeneity of variance (non-Gaussian models). Conditional R^2^ (R^2^_c_) values were calculated on global models [45] to asses overall fit of the model candidate set. Methods concerning the inclusion of variables in the global models are provided in S1 File, with descriptions of the variable codes in Table 1.

**Table 1:** Description of variables used in the global models.

Variable	Data type	Description
lnBW	ordinal	*ln* body weight (kg), offspring range -0.967–2.526, adult range 2.542–3.401
lnBL	ordinal	*ln* body length (cm), offspring range 0.478–4.220, adult range 4.241–4.489
year	categorical	year, 14 levels
day	ordinal	day of year, range 75–325
age	ordinal	minimum age, range 3–14 (ages ≥ 14 were combined)
sex	binomial	sex
preg	binomial	female reproductive status (1 = pregnant, 0 = not pregnant)
kage	binomial	*age* is minimum age (0) or known age (1)
maxage	ordinal	maximum age at which an individual was measured, range 2–15
death	binomial	Individual died in the same year or the year following the last measurement of body weight and length
TQ_4_	ordinal	Territory quality, range 1–4 (see S1 File)
RP	binomial	offspring detected post emergence (1) or not (0) each year
RS	ordinal	number of offspring produced each year, range 0–4
mother	categorical	identity of mother, 39 levels
mmage	ordinal	minimum maternal age, range 2–15
rain	ordinal	rainfall (mm) in the previous Aug–Oct, range 164–515
RPY	binomial	*RP* in the same territory in the previous year
TE	ordinal	Trap effort in the current and previous year, range 2–51
mmage39	ordinal	minimum maternal age (≥3 years) where ages ≥9 were combined into one age, range 3–9
seasonage	categorical	Offspring age: first year summer (Jul-Aug); first year autumn (Sept-Nov); second year spring (Mar-May); second summer (Jun-Aug), 4 levels
LS	ordinal	Litter size (1, 2, ≥3 offspring)
rainbin	binomial	total rain Apr-Sept in birth year: < 570mm (-1), ≥ 570mm (+1)

#### Somatic senescence

We used data from 93 individuals (45 female and 48 male), each sampled on 2–8 occasions (mean = 3.29, thus 306 observations), to investigate the relationship between age and body weight (controlling for body length and time of year) for sexually mature, full-grown beavers (age ≥ 3 years); using a LMM, with the function *lme* in the R package *nlme* (v. 3.1–102, [46]). Of these, 20 (8 female and 12 male) were of known age and 35 (including 7 of known age) died in the same year, or the year following the last measurement of their body weight and length. We did not include our measure of resource history (*TQ*_*4*_) because this was either unknown or changed during the life of the majority of individuals. We included a random intercept for individual, with a continuous AR(1) correlation structure to account for repeated measures within individuals.

Our global model (Table 1; S1 File) was:

lnBW=lnBL+year+day+age+age2+sex+preg+kage+maxage+death+age×sex+age×kage+age×maxage+age×death+age2×sex+age2×kage+age2×maxage+age2×death

#### Factors influencing senescence in female reproductive output

We estimated annual female breeding success over 36 territories, 25 with *TQ*_*4*_ indices. We observed 65 litters totalling 106 offspring. Due to accession of the dominant breeding position, our analyses included 39 dominant (territory holding) females (minimum age 2–15) observed over 169 occasions. We included individuals where age was not known at the first observation in all models of reproductive output.

#### Reproduction per year (RP)

We used a GLMM, with a binomial error structure and a logit-link function, to investigate the probability that a dominant (territory holding) female reproduced, or not (binary response), in a given year (*RP*), noting potential for a quadratic (non-linear) relationship between *RP* and minimum maternal age. While variation in group sizes could modify the effects of territory quality (*TQ*_*4*_), group size within each territory was highly dependent on reproductive success in the previous year (*RPY*, S1 File) and was therefore not included in the model. Due to the smaller sample of breeding females compared with individuals used in the model of somatic senescence, we removed *kage* from the global model to avoid problems arising from a low ratio of sample size to predictors. We included random intercepts for *mother*. The global model was (Table 1; S1 File):

$$RP\;(0,1)=TE+mmage+mmage2+rain+{TQ}_{4}+RPY+maxage+mmage\times rain+mmage\times {TQ}_{4}+mmage\times RPY+mmage\times maxage$$

#### Reproductive success (RS)

To scrutinise the relationship between maternal age and reproductive output, we repeated this analysis, replacing the binary response of *RP* with the number of offspring produced by each dominant female per year (i.e., reproductive success, *RS*). Litter size ranged from 0–4, leading to a Poisson distribution and a log-link function in the model. The predictors included in the global model were otherwise identical, and we again included random intercepts for *mother*.

#### Litter size (LS)

To investigate the relationship between maternal age and litter size (*LS*), limited to the instances when females bred successfully, we constructed a GLMM with a Poisson error structure and a log-link function, specifying a random intercept for *mother*, and included *TE* as a control variable. Furthermore, we included measures of resources as used in models of *RP* and *RS*. The global model was therefore:

$$LS=TE+mmage39+mmage392+rain+{TQ}_{4}+mmage39\times rain+mmage39\times {TQ}_{4}$$

Where *mmage39* is maternal minimum age class ranging 3–8 and ≥9 years (S1 File).

#### Offspring quality

Given that longer body-length beavers (*BL*) are more likely to achieve dominant breeding positions in this population [38], we assessed the effect of maternal age, litter size and resources (rainfall and territory quality) on (i) individual offspring body weight (*BW*, relative to body length and age–as an index of somatic condition, able to decrease as well as increase) and (ii) individual offspring *BL* (as an index of skeletal size, stabilising at maturity), using a LMM with a Gaussian error structure with an identity link function. These data were collected for 80 offspring (63 measured as kits and 17 as yearlings) in 53 litters from 26 mothers in 24 territories. We included a random intercept for mother. These global models included two-way interaction terms between maternal age and all other predictors except control variables (Table 1; S1 File):

$$lnBW=lnBL+seasonage+LS+mmage39+mmage392+rainbin+{TQ}_{4}+mmage39\times LS+mmage39\times rainbin+mmage39\times {TQ}_{4}$$

And:

$$lnBL=seasonage+LS+mmage39+mmage392+rainbin+{TQ}_{4}+mmage39\times LS+mmage39\times rainbin+mmage39\times {TQ}_{4}$$

### Ethics statement

The study, including all handling and tagging procedures (for details see above), was reviewed and approved by the Norwegian Directorate for Nature Management (permit id 2012/1191, id 2008/14367, id 05/9639, id 04/9793, id 04/334, id 2002/6758, id 2001/7552, id 2002/841, id 2001/635, id 99/1880) and the Norwegian Experimental Animal Board (id 2579, id 2170, id742, id 2005/48612, id 2004/14671, id S-32/03 id S-168-01), which also granted permission to conduct fieldwork in our study area. The owners of the land gave permission to conduct the studies on their properties.

## Results

### Somatic senescence

**Fig 1:**
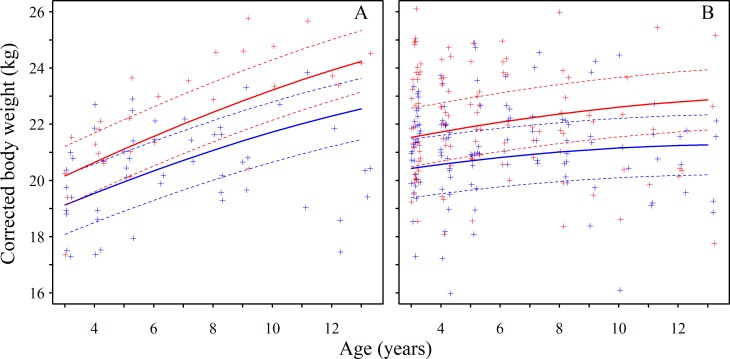
Body condition of adult beavers increased continuously with age. The relationship between age and body weight (controlling for body length and time of year) of beavers ≥ 3 years old where exact age is known (A), or only minimum age is known (B). Lines represent predictions (thick lines) and their 95% prediction intervals (PIs, thin lines) averaged from the top set of LMM models. Points depict raw data. Ages 13–16 were combined. Data pertaining to males are coloured blue and females red.

We found no evidence for somatic senescence in beavers; instead, once fully grown, body weight (*BW*, controlling for body length and season) increased continuously with age, although the rate of increase abated (Table 2; Fig 1). Although males exhibited significantly lower *BW* than females, they did not show a different pattern of weight increase compared to females (non-significant negative *age* × *sex* interaction, Table 2; Fig 1; retained in 16 of the 21 most supported models (total Akaike weights = 0.493, Table 3). R^2^_c_ for the global model was 0.559. The null model had a ΔAICc of +129.3. Although our sample size declined with age (mean 50 individuals per age class from minimum age 3–6 versus mean 12 individuals minimum age 10–13), the mean and SD of *BW* did not change markedly (21.7 ± 1.1 kg and 21.2 ± 1.1 kg in the 3–6 and 10–13 minimum age classes respectively).

**Table 2:** Model estimates of effects on adult body weight.

		95% CI			
	Estimate	lower	upper	*t*	
Intercept	-1.25	-2.32	-1.79	-2.24	*
Day-of-year	3.63E-04	2.21E-04	5.05E-04	5.10	*
lnBL	0.982	0.739	1.226	7.94	*
Sex (male)	-5.72E-02	-8.75E-02	-2.70E-02	-3.84	*
Known age	-3.26E-02	-7.44E-02	-0.93E-02	-1.48	*
Age	0.112	-0.100	0.324	1.83	
Age^2^	-3.79E-02	-10.4E-02	2.80E-02	-2.17	
Pregnant	0.64E-02	-1.83E-02	3.10E-02		
Max age	1.93E-03	-4.61E-03	8.45E-03		
Died	-0.89E-02	-3.97E-02	2.19E-02		
Age × sex (male)	-2.18E-02	-6.02E-02	1.66E-02	-2.03	
Age × known age	0.154	0.025	0.282	2.96	*
Age × max age	-0.20E-02	-2.39E-03	1.99E-02		
Age^2^ × sex (male)	-0.06E-02	-2.01E-02	1.88E-02		
Age^2^ × known age	-2.47E-02	-4.73E-02	-0.21E-02	-2.20	*
Age^2^ × max age	4.28E-03	-0.90E-03	9.45E-03		

Model averaged estimates for LMM models describing *ln* body weight (kg) of beavers ≥3 years old. Estimates where the 95% confidence intervals do not include zero (indicating significance) are marked with an asterisk. Values of t are included from the single most supported model. Not all variables in the top model set were in this model.

**Table 3:** Model selection tables examining the effects on body weight, controlling for body length and time of year.

Inter-cept	*age*	*age2*	*kage*	*inBL*	*maxage*	*died*	*preg*	*sex*	*day*	*year*	*age× kage*	*age× maxage*	*age× sex*	*age2× kage*	*age2× maxage *	*age2× sex*	AICc	ΔAICc	Akaike weight
-1.20	0.062	-0.015	-0.030	0.972				+	3.63E-04	+	0.171		+	-2.53E-02			-693.1		0.082
-1.25	0.234	-0.079	-0.031	0.985	1.13E-03			+	3.69E-04	+	0.166	-9.30E-03	+	-2.53E-02	4.37E-03		-692.9	0.23	0.073
-1.33	0.047	-0.027	-0.032	0.997	3.03E-03			+	3.63E-04	+	0.160	6.73E-03	+	-2.37E-02			-692.3	0.82	0.054
-1.23	0.052	-0.015	-0.030	0.979				+	3.64E-04	+	0.164			-2.49E-02			-691.3	1.83	0.033
-1.17	0.064	-0.015	-0.030	0.965		+		+	3.65E-04	+	0.171		+	-2.52E-02			-691.1	2.05	0.029
-1.19	0.063	-0.015	-0.030	0.970			+	+	3.61E-04	+	0.167		+	-2.46E-02			-691.1	2.06	0.029
-1.21	0.241	-0.080	-0.031	0.978	1.16E-03	+		+	3.71E-04	+	0.166	-9.81E-03	+	-2.52E-02	4.48E-03		-690.9	2.27	0.026
-1.21	0.062	-0.015	-0.031	0.974	6.64E-04			+	3.64E-04	+	0.171		+	-2.52E-02			-690.8	2.36	0.025
-1.20	0.060	-0.014	-0.030	0.973				+	3.64E-04	+	0.170		+	-2.52E-02		+	-690.7	2.41	0.024
-1.24	0.235	-0.079	-0.032	0.983	1.09E-03		+	+	3.67E-04	+	0.163	-9.35E-03	+	-2.47E-02	4.36E-03		-690.6	2.48	0.024
-1.36	0.037	-0.027	-0.032	1.004	2.89E-03			+	3.63E-04	+	0.152	6.66E-03		-2.32E-02			-690.4	2.69	0.021
-1.25	0.233	-0.079	-0.031	0.985	1.12E-03			+	3.69E-04	+	0.166	-9.33E-03	+	-2.52E-02	4.38E-03	+	-690.4	2.70	0.021
-1.19	0.101	-0.023	-0.048	0.974				+	3.45E-04	+	0.047		+				-690.3	2.80	0.020
-1.30	0.199	-0.072	-0.031	0.994	1.25E-03			+	3.68E-04	+	0.157	-7.24E-03		-2.44E-02	3.78E-03		-690.3	2.84	0.020
-1.31	0.048	-0.027	-0.032	0.992	3.10E-03	+		+	3.64E-04	+	0.159	6.66E-03	+	-2.36E-02			-690.1	2.99	0.018
-1.33	0.048	-0.027	-0.033	0.995	2.99E-03		+	+	3.61E-04	+	0.156	6.64E-03	+	-2.32E-02			-690.1	3.04	0.018
-1.34	0.082	-0.036	-0.050	1.002	3.45E-03			+	3.45E-04	+	0.043	7.11E-03	+				-690.0	3.12	0.017
-1.26	0.251	-0.082	-0.050	0.990	1.78E-03			+	3.49E-04	+	0.042	-7.20E-03	+		3.91E-03		-690.0	3.16	0.017
-1.34	0.047	-0.027	-0.032	0.997	3.03E-03			+	3.63E-04	+	0.160	6.73E-03	+	-2.37E-02		+	-689.8	3.28	0.016
-1.20	0.053	-0.015	-0.030	0.973		+		+	3.65E-04	+	0.163			-2.47E-02			-689.2	3.95	0.011
-1.23	0.053	-0.015	-0.030	0.977			+	+	3.62E-04	+	0.160			-2.42E-02			-689.2	3.97	0.011

Due to our estimate of inferred age tending to over-estimate (but not under-estimate) age, *BW* of known age individuals was initially lower than those of inferred-age, with a later slowing in *BW* rate increase (significant negative *age*^*2*^ × *kage* interaction, Table 2; Fig 1). *Age*, *age*^*2*^, *kage* and the *age × kage* interaction were retained in all 21 of the most supported models (Akaike weights = 0.589) while the *age*^*2*^ *× kage* interaction was retained in 18 of the most supported models (Akaike weights = 0.535, Table 3). Proximity to death did not influence the detection of somatic senescence, with the *age × death* and *age*^*2*^ *× death* interactions not retained in any of the most supported models. Pregnancy status did not influence female body weight (Table 2) and was retained in only four of the most supported models (Akaike weights = 0.082).

The lack of a significant positive interaction between *maxage* and *age* (retained in 10 of the most supported models, Akaike weights = 0.424, Table 2; Table 3), or *age*^*2*^ (retained in 5 models, Akaike weights = 0.233) implied that this increase in *BW* occurred at the individual, and not at the population level, i.e., not due to differential survival (ST).

### Senescence in female reproductive output

#### Reproduction per year (RP)

*RP* was influenced by a quadratic effect of maternal minimum age (*mmage*^*2*^), a negative effect of *rain*, positive effect of *TQ*_*4*_ and a positive *mmage* × *TQ*_*4*_ interaction. All of these predictors, except *mmage*^*2*^, were retained in each of the top seven models (ΔAICc <4) (Table 4, Akaike weights = 0.534), where 95% CIs did not span zero (Table 5). Models including *mmage*^*2*^ comprised six of the top models (Akaike weights = 0.480). *RPY*, *maxage* and the interactions of *RPY*, *rain* and *maxage* with *mmage* were retained in fewer of the top models (Table 4); 95% CIs indicated non-significance (Table 5). R^2^_c_ for the global model was 0.512 and the null model had a ΔAICc of +23.6.

**Table 4:** Model selection for probability of reproduction and reproductive success.

Response	Intercept	*TE*	*mmage*	*mmage*^*2*^	*rain*	*RPY*	*TQ*_*4*_	*maxage*	*mmage × rain*	*mmage × RPY*	*mmage × TQ*_*4*_	*mmage × maxage*	AICc	ΔAICc	Akaike weight
Probability of reproduction (0,1)	-0.042	0.170	2.642	-0.666	-0.511		0.747				0.664		206.7	0.00	0.189
	-0.049	0.144	2.535	-0.658	-0.510		0.750	0.109			0.673		208.7	2.04	0.068
	-0.121	0.166	2.582	-0.653	-0.525	0.161	0.729				0.643		208.7	2.04	0.068
	-0.049	0.160	2.631	-0.671	-0.540		0.749		-0.112		0.672		208.7	2.06	0.068
	-0.177	0.200	-0.162		-0.497		0.712	-0.278			0.573	-0.707	209.2	2.51	0.054
	-0.052	0.157	2.761	-0.641	-0.485	-0.022	0.800			-0.624	0.737		209.3	2.66	0.050
	-0.032	0.176	1.640	-0.425	-0.518		0.731	-0.117			0.643	-0.392	209.9	3.25	0.037
														*Total*	*0*.*534*
Reproductive success (N offspring)	-0.402	0.102	1.401	-0.343	-0.326		0.372				0.274		211.0	0.00	0.096
	-0.380	0.115	1.347	-0.333	-0.334		0.261						211.6	0.60	0.071
	-0.404	0.081	1.269	-0.331	-0.322		0.365	0.159			0.282		212.3	1.37	0.048
	-0.374	0.111	1.606	-0.352	-0.294	-0.114	0.426			-0.485	0.338		212.7	1.71	0.041
	-0.382	0.097	1.222	-0.321	-0.330		0.252	0.145					213.1	2.11	0.033
	-0.405	0.098	1.380	-0.342	-0.333		0.369		-0.040		0.273		213.1	2.16	0.032
	-0.403	0.101	1.400	-0.343	-0.326	0.002	0.372				0.274		213.2	2.23	0.031
	-0.389	0.120	1.537	-0.370	-0.332								213.4	2.39	0.029
	-0.384	0.111	1.323	-0.331	-0.341		0.259		-0.045				213.7	2.71	0.025
	-0.400	0.111	1.331	-0.329	-0.339	0.050	0.256						213.7	2.75	0.024
	-0.485	0.129	-0.102		-0.323		0.381	-0.015			0.243	-0.345	214.0	3.02	0.021
	-0.446	0.139	-0.096		-0.333		0.269	-0.028				-0.364	214.0	3.05	0.021
	-0.372	0.119	1.498	-0.339	-0.318	-0.037	0.277			-0.365			214.4	3.37	0.018
	-0.394	0.091	1.027	-0.267	-0.326		0.361	0.097			0.273	-0.118	214.4	3.39	0.018
	-0.395	0.097	1.361	-0.352	-0.329			0.189					214.4	3.40	0.017
	-0.372	0.098	1.503	-0.344	-0.291	-0.115	0.418	0.119		-0.453	0.340		214.5	3.54	0.016
	-0.408	0.077	1.244	-0.330	-0.329		0.363	0.159	-0.043		0.280		214.5	3.55	0.016
	-0.401	0.082	1.272	-0.332	-0.321	-0.008	0.366	0.159			0.283		214.6	3.62	0.016
	-0.684	0.146	-0.180		-0.305		0.443				0.259		214.8	3.81	0.014
	-0.367	0.109	0.870	-0.229	-0.336		0.248	0.064				-0.167	214.8	3.85	0.014
	-0.366	0.115	1.638	-0.355	-0.287	-0.126	0.431		0.031	-0.499	0.343		214.9	3.96	0.013
														*Total*	*0*.*614*

Model selection results for GLMM model sets describing probability of reproduction (binomial) and reproductive success (number of offspring, Poisson). Only models within AICc 4 of the top model are included. Trap effort was included in all candidate models within each set.

**Table 5:** Model estimates on the probability of reproduction and reproductive success.

Response:	Probability of reproduction (0,1)					Reproductive success (N offspring)				
		95% CI					95% CI			
Predictor	Estimate	lower	upper	*z*		Estimate	lower	upper	*z*	
Intercept	-0.068	-0.596	0.461	-0.17		-0.403	-0.762	-0.044	-2.41	*
Trap effort (*TE*)	0.167	-0.196	0.530	0.94		0.106	-0.114	0.326	0.93	
Maternal age (*mmage*)	2.278	-0.599	5.154	2.17		1.228	-0.366	2.822	2.01	
*mmage*^*2*^	-0.642	-1.206	-0.079	-2.44	*	-0.335	-0.638	-0.031	-2.27	*
*rain*	-0.513	-0.889	-0.136	-2.71	*	-0.324	-0.551	-0.097	-2.86	*
*RPY*	0.084	-0.690	0.857			-0.048	-0.501	0.405		
*TQ*_*4*_	0.746	0.290	1.201	3.26	*	0.338	0.021	0.656	2.42	*
*maxage*	-0.075	-0.736	0.586			0.111	-0.277	0.499		
*mmage × rain*	-0.112	-0.653	0.429			-0.031	-0.351	0.288		
*mmage × RPY*	-0.624	-1.591	0.344			-0.457	-1.049	0.135		
*mmage × TQ*_*4*_	0.660	0.094	1.226	2.34	*	0.286	-0.056	0.627	1.60	
*mmage × maxage*	-0.578	-1.301	0.144			-0.262	-0.723	0.199		

Model averaged results from the top (ΔAICc <4) set of GLMM models describing probability of reproduction (binomial) and reproductive success (number of offspring; Poisson). Estimates where the 95% confidence intervals do not include zero (indicating significance) are marked with an asterisk. Values of z are included from the single most supported model for each. Not all variables in the top model set were in these models.

**Fig 2:**
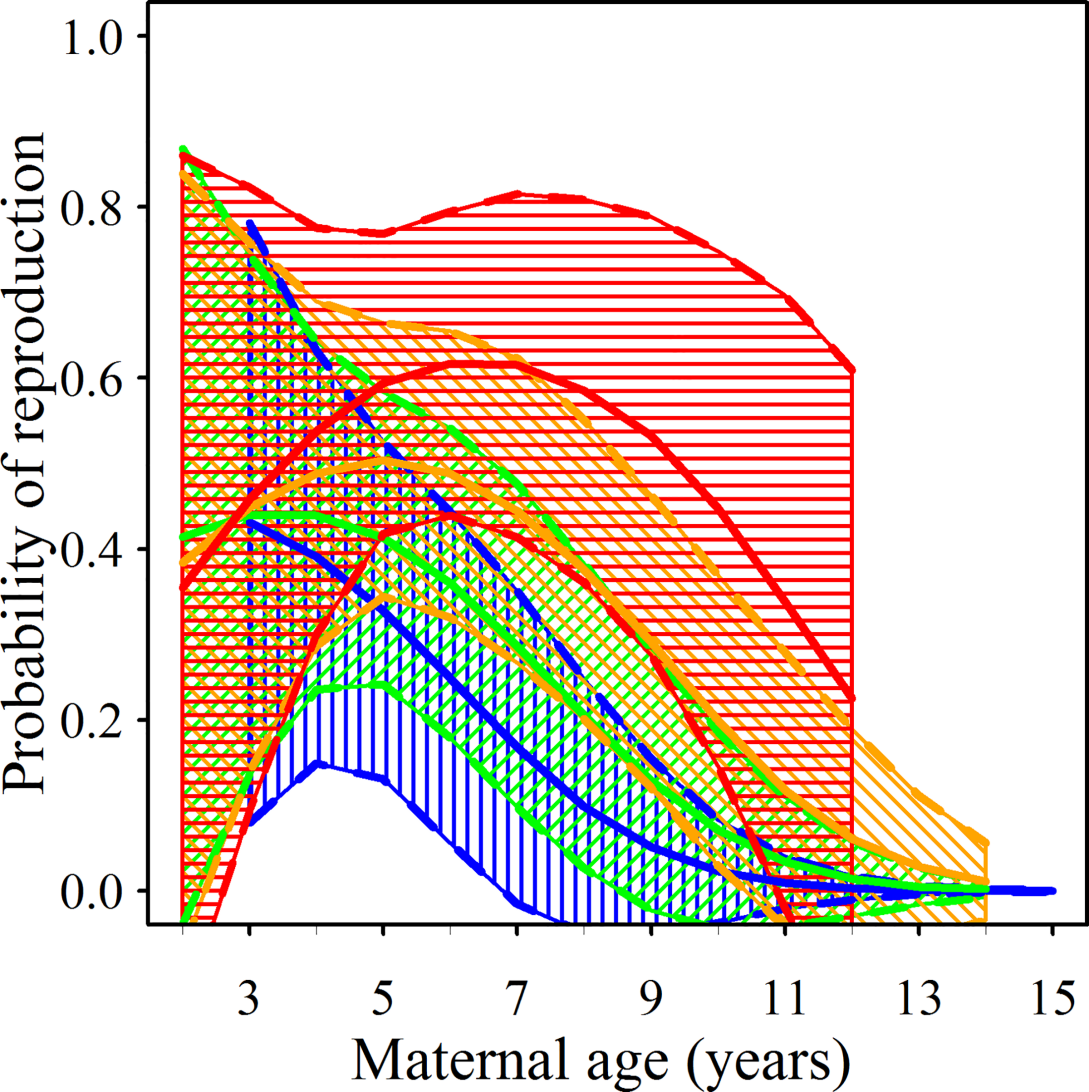
Adult females from low quality territories are significantly less likely to reproduce as they age. The relationship between probability of reproduction and maternal minimum age (*mmage*) for the four levels of territory quality (*TQ*_*4*_). Colours denote *TQ*_*4*_ where blue = 1, green = 2, orange = 3 and red = 4. Lines present predictions and shading denotes areas within the 95% prediction intervals, averaged from the top set of GLMM models.

These models (Fig 2, see S1 Fig for plots of the response surface, sample size and mean values) imply that a substantial reduction in *RP* occurred with age, particularly in lower quality (*TQ*_*4*_ < 4) territories (e.g., for *TQ*_*4*_ = 1, upper prediction intervals at *mmage* 10 are lower than lower prediction intervals at *mmage* four).

A decline in *RP* with age for *TQ*_*4*_ = 4 was less apparent; the peak prediction interval was at the minimum age six (0.44–0.80), compared to at minimum age 12 (0–0.61). For *TQ*_*4*_ > 1, *RP* was also lower for younger animals, but again this was non-significant (e.g., when *TQ*_*4*_ = 4 and *mmage* = 2, *RP* = 0–0.86). Wide prediction intervals for the youngest mothers might relate to a smaller sample size.

Overall, with lower *TQ*_*4*_, we observed a lower peak in *RP*, which also occurred at a younger age. Consequently, from minimum age six, mothers in territories with *TQ*_*4*_ = 1 were significantly less likely to produce offspring compared to those occupying highest quality territories (*TQ*_*4*_ = 4). Moving from the highest to lowest quality territories, model predictions indicate the highest probability of reproduction was at minimum ages 6–7, 5, 3–4 and 3 years (Fig 2).

From the 39 mothers examined, sample sizes were smaller for older age classes with a mean sample size per age class of seven individuals over minimum ages 8–15, versus a mean of 20 individuals over minimum ages 2–7. Nevertheless, the general reduction in *RP* with age was clear (44% bred per year over minimum ages 2–7 and 25% over minimum ages 8–15) while five (13%) survived for ≥3 years (mean = 6 years ± 3.6 SD, range = 3–11) after their last successful reproductive event.

#### Reproductive success

Modelling *RS* (number of offspring recorded for each dominant / breeding female in each year) in place of *RP* changed the biological interpretation of the model selection process somewhat (Table 4). *Mmage* and *rain* contributed to all of the 21 top models (total Akaike weights = 0.614, Table 4), *TQ*_*4*_ contributed to 19 (Akaike weights = 0.568), while *mmage*^*2*^ contributed to 18 (Akaike weights = 0.558). The *mmage* × *TQ*_*4*_ interaction contributed to 12 of the top models (Akaike weights = 0.362 Table 4) but was marginally non-significant (CI = -0.056–0.627, Table 5). Averaging these model estimates reaffirmed a negative effect of *mmage*^*2*^, a negative effect of *rain*, and a positive effect of *TQ*_*4*_ on reproductive success (Table 5). The *mmage* and *mmage*^*2*^ estimates suggest that peak *RS* (assuming all other variables are at their mean or median) occurred at minimum age five, when mean age-specific *RS* = 0.75 offspring / year (95% CIs = 0.35 to 1.14). R^2^_c_ for the global model was 0.505 and the null model had a ΔAICc of +184.

#### Litter size (LS)

Mean litter size over the study period was 1.66 ± 0.84 SD. We found no effect of individual maternal age (*mmage39*) or of either resource proxy (*rain* and *TQ*_*4*_) on *LS*, on occasions when females bred successfully; with greatest support for the null model (containing only *TE*, Akaike weight = 0.325). R^2^_c_ for the global model was 0.407.

Models including *mmage39* had less influence (three of the six top models, total Akaike weights = 0.220). Similarly, there was little support for models including *rain* (two of the top models, Akaike weights = 0.170) or *TQ*_*4*_ (two of the top models, Akaike weights = 0.155), while there was scant support for the models that also included *mmage39*^*2*^ and interactions of *mmage39*×*rain* and *mmage39*× *TQ*_*4*_ (all outside the top model set with ΔAICc > 4). Averaging from the top models confirmed no effect of *mmage39* (estimate = 0.066, 95% CIs = -0.129 to 0.261), *rain* (estimate = -0.085, 955% CIs = -0371 to 0.202) or *TQ*_*4*_ (estimate = -0.040, 95% CIs = -0.249 to 0.169).

#### Offspring quality

We found little support for any influence of *mmage39* or *TQ*_*4*_ on offspring *BW*: of the 14 top models with a ΔAICc < 4 (total Akaike weights = 0.790), *mmage39* contributed in seven (Akaike weights = 0.252) and *TQ*_*4*_ in four (Akaike weights = 0.164). This was corroborated by model averaged estimates: *mmage39* estimate -0.023, 95% CIs = -0.105 to 0.058; *TQ*_*4*_, estimate = -0.023, 95% CIs = -0.081 to 0.034. Apart from the control variables (*lnBL* and *seasonage*), only *rainbin* (‘dry’ versus ‘wet’ years) effected offspring *BW*, with a negative influence (estimate = -0.045, 95% CIs = -0.087 to -0.003); where *rainbin* contributed to 10 of the top models (Akaike weights = 0.581). Litter size (*LS*) contributed to seven of the top models (Akaike weights = 0.459), although the effect was not quite significant (estimate = -0.039, 95% CIs = -0.080 to 0.001). R^2^_c_ for the global model was 0.907 and the null model displayed a ΔAICc of +12.8.

**Fig 3:**
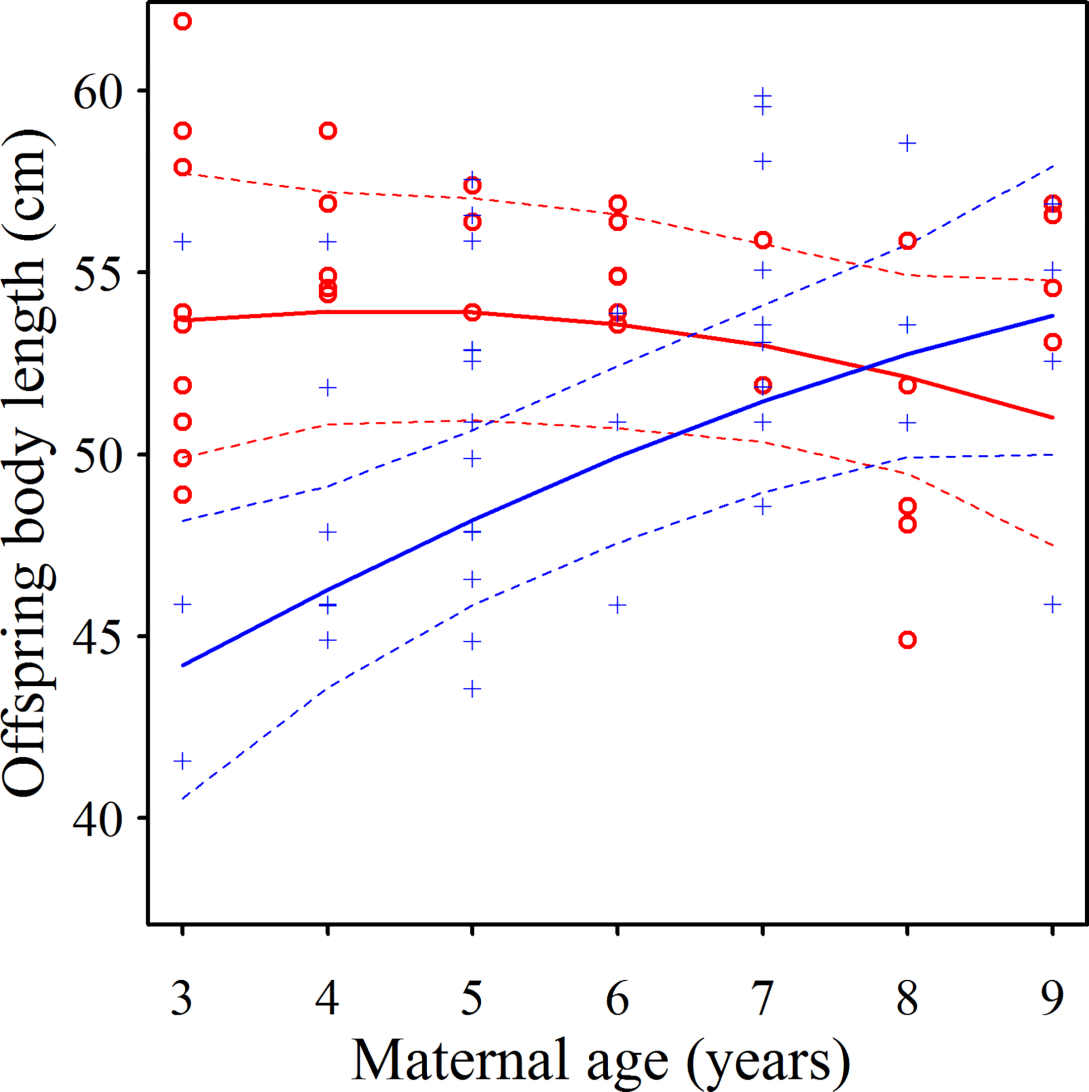
During resource poor years offspring from younger mothers are smaller. The relationship between offspring body length and maternal minimum age in dry (‘○’, red) and wet (‘+’, blue) years. Data points represent raw data (body length controlling for age) while lines provide predictions and 95% prediction intervals derived from the averaged top set of LMM models.

**Fig 4:**
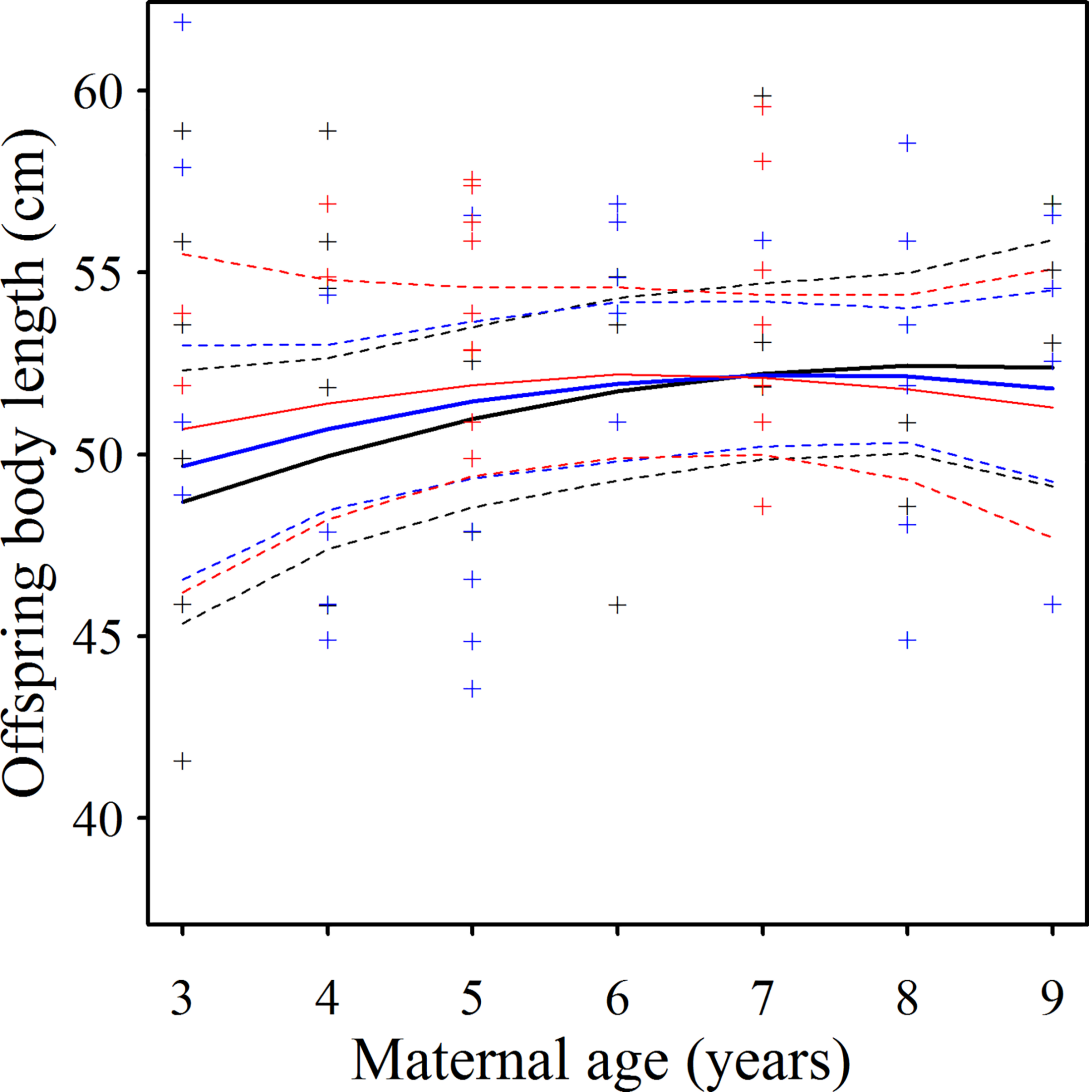
Offspring size was comparable for all litter sizes irrespective of mother age. The relationship between maternal age 3–8 and ≥9) and offspring body length (BL, cm) at different litter sizes (LS) where black indicates LS = 1; blue LS = 2 and red LS ≥ 3. Solid lines indicate predictions and dashed lines indicate 95% prediction intervals. Predictions are from model averaged estimates of the most supported (ΔAICc < 4) models. The large overlap in prediction intervals suggests that the effects of LS and maternal age are marginal.

Effects on body length (*BL*) of *mmage39*, *mmage39*^*2*^, *LS* and *rainbin* were modified by interactions with *mmage39* with *rainbin* (positive, evident in all 16 top ΔAICc <4 models, Akaike weights = 0.980, Table 6; Table 7) and *mmage39* with *LS* (negative, six top models, Akaike weights = 0.382). R^2^_c_ for the global model was 0.736 and the null model displayed a ΔAICc of +77.4. The predictions of the averaged top models implied that:

1. Offspring *BL* was unaffected by maternal age in drier years, but increased markedly with maternal age in wetter years. Thus, wetter (resource poor) years impaired offspring *BL* only for younger mothers (Fig 3).
2. Offspring *BL*, in litters of one kit, was slightly longer among kits born to older mothers, although the magnitude of the increase was less with larger litter sizes (Fig 4). The effect of maternal age and its interaction with litter size was marginal however, as evidenced by the interaction appearing in fewer of the top models, and exhibiting a large overlap of prediction intervals (Fig 4).
3. Offspring *BL* was unaffected by territory quality, irrespective of the age of the mother.

**Table 6:** Model selection for offspring body weight and body length.

	inter-cept	*lnBL*	*age*	*LS*	*mmage*	*mmage*^*2*^	*rain*	*TQ*_*4*_	*mmage × LS*	*mmage × rain*	*mmage × TQ*_*4*_	AICc	Δ AICc	Akaike weight
*BC (ln BM)*	-5.271	1.790	+	-0.034			-0.039					-48.3	0.0	0.137
	-5.040	1.735	+				-0.051					-48.2	0.1	0.129
	-5.829	1.927	+	-0.047								-47.7	0.6	0.102
	-5.018	1.732	+		-0.025		-0.052					-47.0	1.3	0.073
	-5.234	1.783	+	-0.032	-0.021		-0.041					-46.6	1.7	0.060
	-5.278	1.790	+	-0.037			-0.039	-0.028				-46.5	1.8	0.057
	-5.030	1.731	+				-0.051	-0.022				-46.1	2.2	0.045
	-5.843	1.929	+	-0.049				-0.026				-46.0	2.3	0.043
	-5.820	1.927	+	-0.045	-0.018							-45.8	2.5	0.039
	-5.700	1.898	+									-44.9	3.4	0.025
	-5.215	1.781	+		-0.025		-0.047			-0.009		-44.5	3.8	0.021
	-5.620	1.880	+	-0.035	-0.021		-0.031			-0.016		-44.5	3.8	0.021
	-5.015	1.731	+		-0.023		-0.052	-0.009				-44.4	3.9	0.019
	-5.017	1.732	+		-0.027	0.000	-0.052					-44.3	4.0	0.019
													*Total*	0.790
	3.943		+	0.0117	0.007		-0.040		-0.018	0.037		-160.7	0.00	0.121
*lnBL*	3.938		+		0.011		-0.039			0.036		-160.7	0.08	0.116
	3.956		+		0.158	-0.022	-0.045	-0.020		0.042		-160.5	0.19	0.110
	3.959		+		0.123	-0.018	-0.043			0.040		-160.5	0.24	0.108
	3.958		+	0.0047	0.141	-0.019	-0.043	-0.019	-0.019	0.042		-160.2	0.58	0.091
	3.96		+	0.0066	0.108	-0.016	-0.042		-0.018	0.041		-159.7	1.07	0.071
	3.933		+		0.017		-0.040	-0.015		0.037		-159.4	1.33	0.062
	3.935		+	0.0103	0.016		-0.039	-0.019	-0.020	0.038		-159.3	1.48	0.058
	3.94		+	0.0105	0.011		-0.041			0.036		-159.2	1.54	0.056
	3.956		+	0.0060	0.107	-0.015	-0.044			0.039		-158.1	2.63	0.033
	3.933		+		0.024		-0.040	-0.028		0.037	-0.015	-157.9	2.84	0.029
	3.954		+	0.0037	0.150	-0.021	-0.046	-0.020		0.041		-157.9	2.88	0.029
	3.954		+		0.144	-0.019	-0.044	-0.022		0.041	-0.005	-157.8	2.90	0.028
	3.934		+	0.0098	0.016		-0.042	-0.014		0.037		-157.8	2.97	0.027
	3.936		+	0.0087	0.020		-0.039	-0.024	-0.018	0.038	-0.009	-157.2	3.50	0.021
	3.958		+	0.0047	0.143	-0.020	-0.043	-0.019	-0.019	0.043	0.001	-157.1	3.61	0.020
													*Total*	0.980

Model selection results for LMM model sets describing offspring *ln* body weight (*BW*) and *ln* body length (*BL*). Only models within AICc 4 of the top model are included. Age was included in all candidate models within each set while lnBL was included in all candidate models describing *lnBW* so that *lnBW* equates to body condition (*BC*).

**Table 7:** Model estimates on offspring body weight and body length.

	*lnBW*					*lnBL*				
		95% CI					95% CI			
Predictor	Estimate	lower	upper	*t*		Estimate	lower	upper	*t*	
Intercept	-5.334	-6.983	-3.685	-6.71	*	3.948	3.905	3.990	226	*
*lnBL*	1.807	1.392	2.222	9.01	*					
Age: summer 0	-0.233	-0.345	-0.120	-4.76	*	-0.121	-0.165	-0.077	-5.97	*
Age: spring 1	0.022	-0.091	0.136	0.54		0.137	0.079	0.195	4.57	*
Age: summer 1	0.126	-0.114	0.367	1.19		0.332	0.236	0.428	7.30	*
Age: autumn 1	0.342	0.079	0.606	2.62	*	0.275	0.159	0.392	5.46	*
Litter size (*LS*)	-0.039	-0.080	0.001	-1.73		0.008	-0.010	0.027	1.28	
Maternal age (*mmage*)	-0.023	-0.103	0.058			0.073	-0.079	0.226	0.74	
*mmage*^*2*^	0.000	-0.040	0.041			-0.019	-0.039	0.002		
*Rain*	-0.045	-0.087	-0.003	-1.88	*	-0.042	-0.061	-0.022	-4.04	*
*TQ*_*4*_	-0.023	-0.081	0.034			-0.019	-0.040	0.002		
*mmage × LS*						-0.019	-0.035	-0.002	-2.10	*
*mmage × Rain*	-0.013	-0.049	0.024			0.039	0.021	0.057	4.38	*
*mmage × TQ*_*4*_						-0.008	-0.031	0.016		

Model averaged results from the top (ΔAICc <4) set of LMM models describing ln body weight (*BW*) and ln body length (*BL*). Estimates where the 95% confidence intervals do not include zero (indicating significance) are marked with an asterisk. Values of *t* are included from the single most supported model for each. Not all variables in the top model set were in these models.

## Discussion

While several seminal studies have reported a range of life-history trade-offs with senescence (e.g. [7, 25, 47–54]) our study contributes rare evidence supporting resource-dependent theories of senescence (see S2 File for discussion in relation to other studies on *Castor* spp.). No signal for body weight senescence was apparent for adult beavers, and dominant females that achieved an older age in the population were no less likely to breed in a given year, and did not produce fewer offspring per annum than those that died when younger.

Importantly, the fact that the clear signal of reproductive senescence was not mirrored by somatic senescence lends support to Nussey et al.’s [7] contention that heterochrony is widespread in natural populations (see also Hayward et al. [54]), and counters Williams’s [9] prediction of synchrony in the schedule of senescent traits.

### Age-related changes in body condition and offspring production

#### Somatic senescence

Rather than body weight senescence, (controlling for body length and season) beavers of both sexes from minimum age three exhibited an increase in body weight. This contrasts with several other studies on mammals [53,55,56] that found declines in body weight with age. For example, Alpine marmot (*Marmota marmota*) males exhibited terminal decline in body weight (controlling for season), although females did not show any detectable senescence in body weight [53]. In a detailed cross-species analysis, Nussey et al. [55] also found a significant body-mass decline with age in populations of wild bighorn sheep (*Ovis canadensis*), roe deer (*Capreolus capreolus*) and Soay sheep (*Ovis aries*). While they report that the selective disappearance of light individuals contributed to variation in the body-weights they observed with age following the selection theory (ST [16]), we found no evidence for this effect. Although our sample size declined with age, potentially affecting our ability to detect changes in *BW*, the between-individual variance was similar across age classes and the increase in *BW* was significant even controlling for the maximum age attained, indicating that this occurred at the level of individuals and not from the selective mortality of lower quality animals at younger ages, and so did not accord ST, [16]. We found no evidence that somatic condition declined in the year prior to death, with the 35 individuals that died in the same or following year after measurement exhibiting the same pattern of increasing body weight as those individuals that survived for longer after the last measurement was recorded.

While we do not show here that body weight influenced fitness directly, our previous studies on this same beaver population have shown significant and consistent weather effects on body weight, reproduction and survival probability [26,31], suggesting that body weight is related to fitness. Nevertheless, future studies on this species could examine changes in skeletal density, muscle mass and mass of internal organs in more detail to establish whether the body weight pattern observed here is mirrored across other physical traits.

Given that both sexes (especially both breeding and non-breeding females) exhibited an increase in *BW* with age, we propose that this body-weight result may arise due to foraging experience following the constraints theory (CT [15–17]), with older individuals able to secure food resources more successfully, and was not linked to early-life breeding restraint, which would evidence somatic conservation following the disposable soma and reproductive restraint theories (DS and RR [11–13]).

#### Reproductive investment and age

Reproduction was already close to maximal at primiparity, or at the earliest observation where age was unknown. Although we detected an initial increase in both probability of annual reproduction and reproductive success (hereafter ‘reproduction’) with age for mothers in high quality territories (up to minimum age 5–6 years), this increase was not significant, contrary to CT [15–17].

However, overlaying annual resource effects, we did find that while the offspring born to younger mothers (≤5 years minimum age) during resource-poor years (high rainfall) were significantly smaller (juvenile body length), this was not the case for offspring born to older mothers (≥6). This suggests a greater influence of resource availability on younger mothers, consistent with CT and potentially ST (though see below). Crucially, large adult body size determines whether a beaver gains dominant breeding status and juvenile beavers may prioritise investment in gaining body-length at the expense of body condition [38]. The greater experience [57] or superior body condition of older mothers [58] might influence their capacity to respond to more challenging conditions (but see below), because conversely, in resource rich (dry) years (see [31]), mothers of all ages produced offspring with similar body sizes.

#### Reproductive senescence

That we were able to observe only 31% of dominant females over their entire reproductive life-span inevitably restricts our ability to investigate reproductive senescence fully. Nevertheless, we observed a significant decline in annual reproduction, after minimum age five. This decline in annual reproduction with age is predicted by DS [11] RR, [13], and congruent with other mammalian studies [50,59,60]. The disappearance of faster-breeding or lower quality, and thus shorter-lived individuals from the population (following ST, [16]) does not adequately explain the actuarial pattern of senescence we observed: dominant females that achieved an older age in the population were no more or less likely to breed in a given year, and did not produce greater or fewer offspring per annum, than those that died when younger.

In terms of resource affects, overlain on a general senescent decline in reproduction, reproductive metrics were consistently lower in low quality territories, irrespective of maternal age and annual rainfall. Annual variation in rainfall did not, however, influence the onset of reproductive senescence (the age at which the trait measured begins to show a year-on-year decline) directly, with similar responses for females of all age. Consequently, older (and higher *BW*, see above) mothers responded to short-term (inter-annual) resource variation similarly to younger mothers in terms of reproductive output, despite also exhibiting a senescent decline in reproduction. In contrast, older mothers are less affected by short-term resource variation, as measured by offspring size. This dual response could arise if older mothers accumulate greater energy reserves (accruing capital, *sensu* [61]) during one, or more, barren years, in order to mitigate unexpected poor conditions in the birth year.

#### Reproductive senescence onset

Williams [9] proposed that senescence should begin immediately after maturation (i.e. at the start of the decline in reproductive potential). By contrast, we observed a mismatch between sexual maturity (occurring at age 2–3) and the earliest breeding opportunity. In beavers, only dominant pairs (territory holders, determined by body size, [38]) breed, creating strong reproductive skew, and thus not all beavers begin breeding upon reaching sexual maturity at around age two [28,29]. In our study system territories are acquired at the mean age of five (S1 Fig); the age beyond which senescence in annual reproduction probability also commenced. Indeed, based on a dominant adult annual mortality rate of 13% per annum in this same population [26], 50% of beavers that achieved dominant breeding positions will die before age seven; approximating the age at which reproductive success started to decline. This corroborates Jones et al. [6] who found that for terrestrial vertebrate species in which the mean female reproductive age is 5–7, the relationship between age at onset of senescence (both actuarial and reproductive) and mean reproductive age was close to synchronous.

#### Resource history and onset of senescence

Studies have reported a trade-off between early and late reproduction in species as diverse as flycatcher (*Ficedula albicollis*) [47] and female red deer (*Cervus elaphus*) [60], but without reference to whether this trade-off was mediated by resources, as predicted by DS. In this territorial species, we found that individuals that experienced impoverished resource availability through their reproductive lifespan, measured as territory quality, experienced a relatively earlier onset of annual reproductive senescence (*RP*: litter produced, or not) than individuals in higher quality territories, although reproductive success (*RS*: annual number of offspring produced) was not affected. It is plausible that females in poor quality territories may allocate more absolute resources to reproduction (not just a relatively larger proportion of available resources) than those in high quality territories as a strategy to counter a lower realised longevity.

We could not measure resource allocation directly, only reproductive output; however, the mechanism involved may relate to physiological stress arising from the elevated relative energy expenditure required to raise litters successfully in poor quality territories. This could potentially affect longevity assurance mechanisms [22], induce greater oxidative stress, exacerbate telomere shortening rates [62], or induce apoptosis [63]. Alternatively, following RR [13], this pattern may result from females in poor quality territories reducing reproductive effort due to poor somatic condition, in order to extend their remaining lifespan and take advantage of breeding opportunities in later years. Indeed, this decoupling of somatic condition and reproductive success through reproductive restraint could explain the asynchrony between somatic and reproductive senescence we detected.

Notably, there was no evidence that the link between territory quality and senescence onset could be explained by higher quality individuals residing in better territories (S2 Fig). The difference in the response of *RP* and *RS* could arise if older individuals in lower quality territories are more likely to incur whole-litter breeding failures, rather than partial breeding failures (i.e. resource history and age interact to influence the probability that *RS* = 0 versus ≥1 but not the probability that *RS* = 1, 2 or 3). In support of this, we found no evidence that maternal age or territory quality influenced litter sizes.

These results exemplify state-dependent life-history strategies [64]; that is, age-dependent reproductive profiles differed between individuals depending on individual circumstances. Local resource competition also affects earlier onset and higher rate of physical senescence in male red deer in Norway [65]. Similarly, guillemots (*Uria aalge*) experiencing harsher environmental conditions early in life undergo more rapid senescence [52]. Together, these demonstrate generally that a trade-off between somatic maintenance and reproduction can arise as a direct result of resource limitation (supporting resource-dependent hypotheses of senescence, such as DS).

### Conclusions: The general influence of resources and population structure on senescence

Nussey et al. [7] highlight how life-histories differ substantially depending on the environment in which they are measured, and therefore that patterns of aging cannot be characterised without reference to the specific environmental conditions in which they are also measured. By examining the relationship between reproductive senescence and metrics of long- and short-term environmental quality (resources) from this wild beaver population, we demonstrate (see [52,65]) that resource limitation (i.e. residence in low quality territories over the reproductive life-span), and not just reproductive life-cycle phase, can mediate trade-offs in long-term reproductive cost, exemplifying state-dependent life-history strategies [64]. Under these natural circumstances we establish support for resource-dependent hypotheses of ageing (i.e., DS and RR).

We also emphasise how individual-level effects can influence reproduction and somatic maintenance; for example, the offspring of older individuals were not smaller (shorter body length) in resource-poor (high rainfall) years, whereas those of younger mothers were smaller. Thus the life experience of individuals may relate to their ability to acquire resources to invest in somatic maintenance and reproduction, especially in resource-poor years (see [66,67]).

Under circumstances where only territory holding pairs breed, and low turnover in these pairs limits recruitment, the retention of non-breeding philopatric offspring can arise [27]. Where environmental conditions are stable, as in this study (*c*.*f*. [68]), and annual mortality rate is low (here ≤36% for philopatric adults, [26]) this can result in reproductive decline prior to achieving breeding status. Termed the ‘Florida effect’ [68], this phenomenon describes progressively older breeding individuals holding territories, and aged individuals still waiting for a breeding opportunity. As a consequence, adaptive life-history schedules would appear to play a crucial role in population dynamics, where under conditions of limited territory availability, selective pressure drives delayed senescence in order to enhance the life-time reproductive success of individuals that begin breeding late in life, due to a lack of territories. Early breeding opportunities presented to individuals arsing from the background mortality of incumbent territory holders could, however, result in a forward shift in the individuals’ ageing schedule arising from resource dependent senescence mechanisms. We suggest that necessity for flexible life-history strategies to maximise fitness under varying social and environmental conditions might explain the heterochrony in senescence traits observed here and in other studies [7].

## Supporting information

S1 File Additional statistical details. Methods concerning the inclusion of variables in the global models.
(DOCX)

S2 File Comparison with other studies on beaver. (DOCX)

S3 File Data used in the analysis of somatic senescence. (CSV)

S4 File Data used in the analysis of reproductive senescence with reference to probability of reproduction and reproductive success. (CSV)

S5 File Data used in the analysis of reproductive senescence with reference to offspring quality. (CSV)

S1 Fig Effect of maternal age and territory quality on reproduction. Surface plot of the interaction between maternal age, territory quality and probability of reproduction.
(DOCX)

S2 Fig Individual quality and territory quality. (DOCX)
