# Supplementary material for: Age-related changes in somatic condition and reproduction in the Eurasian beaver: Resource history influences onset of reproductive senescence
Source: PLoS One. 2017 Dec 5;12(12):e0187484. doi: 10.1371/journal.pone.0187484 (PMC5716577; doi:10.1371/journal.pone.0187484)
Supplement: S1 File — Methods concerning the inclusion of variables in the global models. (DOCX) [file pone.0187484.s001.docx]

## S1 File. Methods concerning the inclusion of variables in the global models

### Defining territory quality

We quantified the availability of small trees (1≤cm≤5 diameter at 20cm height), which is the preferred size of trees felled by beavers in our study site (Haarberg and Rosell 2006), to provide a metric of the resources available within each territory, where woody vegetation represents the largest proportion (78%) of the diet [27]. Dominant small tree species exhibited no simple pattern of species availability, thus to reduce the ratio of explanatory variables to sample size, data on species was not included in analyses.

Beavers foraged up to ca. 40m inland (Parker et al. 2010; Haarberg and Rosell 2006) and therefore we defined foraging ranges as extending 40m up the riparian bank. We interpolated territory borders from a combination of (a) radio-telemetry (see [27,35]); (b) behavioural observation of animal locations; (c) observations of territorial behaviour, such a scent-marking (Rosell and Thomsen 2006); and (d) surveys of beaver scent-marking mounds, clustered at borders (Rosell et al. 1998). Deciduous habitat blocks ≥10m wide, within 40m of the shore, were delineated into polygons from 1:5000 field maps, and then further divided into sections comprised by similar tree species and structure. Habitat blocks were digitized in 2D (GIS: ArcView 3.x or ArcGIS 9.1, ESRI).

We established typical small tree availability in each habitat block that contained deciduous vegetation by surveying transects along the Sauar river in June-July 2006 and 2007 and the Gvarv and Straumen rivers in June-July 2000. Transects were conducted at 200m intervals perpendicular to the river bank, commencing 50m within the downstream border of each block, or at the middle of the block if the block extended <60m along the river edge. Each transect included four circular points of 5m radius at 5, 15, 25 and 35m distance from the shoreline, where we estimated the percentage cover of small trees 1≤m≤5 in height.

We defined territory quality (*q*) as:

Where *c_i_* represents the mean percentage cover of small trees and *ha_i_* the area in hectares of the *i*th habitat block in each territory.

Based on this metric for territory quality, territories were divided into four categories (TQ_4_): 1, (lowest quality) *q* < 139, N = 6; 2, *q* = 139 – 231, N = 7; 3, *q* = 232 – 390, N = 7; and 4 (highest quality) *q* > 390, N = 8.

### Statistical analyses

Somatic senescence

To model body weight (*BW*), which typically increases from spring through to autumn, we constructed a linear model with *ln* body weight (*lnBW*) as the response variable and *ln* body length (*lnBL*) as a predictor (following Freckleton 2002), along with the factors ‘*year*’ and ‘*day*’, because time of year and annual variation in resources could influence *BW*. We then added a series of other predictors to this model: minimum age (*age*); its quadratic component (*age^2^*); *sex*; female reproductive status (pregnant or not) in the year measured (*preg*), a binary variable (*kage*) that defined whether the individual was of minimum age (0) or known age (1) and a binary variable (*died*) that defined whether the individual died in the same year or the year following the last measurement of body weight and length.

Because the pattern of senescence might vary between sexes, we specified a *sex* × *age* and a *sex* × *age^2^* interaction. To model unknown age versus assigned minimum age, we included a *kage* × *age* and a *kage* × *age^2^* interaction term. Failure to detect a senescent decline in somatic condition in later life might be due to this decline only occurring as the individual neared death. We therefore included a *died* × *age* and a *died* × *age^2^* interaction term. Establishing, at the population level, that body weight typically increases with age (see results), we examined whether this effect could arise simply because poor quality (lower body condition) animals disappeared from the population earlier, due to differential mortality rate (ST). We thus added the maximum age at which an individual was measured (*maxage*), and a *maxage* × *age* and a *maxage* × *age^2^* interaction to the model. ST would predict a positive interaction (individuals that only achieve a lower *maxage* would show a smaller effect of age on the increase in *BW*). *Age* was centred at zero mean (mean = 5.30) and unit variance (SD = 3.04).

We included a random intercept for individual (random intercept model, Schielzeth and Forstmeier 2009) and specified a CAR1 autocorrelation structure for random effects, since body condition at times *t* and t*+1* could be correlated. We categorized all individuals > 13 years as elderly; two individuals survived to 14 (both female), one male to 15 and one female to 16.

From the global model (see main text), we specified a subset of candidate models including all possible combinations of fixed effects, except that *lnBL* and *day* were retained in all models, and the inclusion of *age^2^* was conditional on the inclusion of *age*.

Senescence in female reproductive output

Previous research on the same population also found a significant negative effect of rainfall in the preceedings late-summer and autumn (Aug-Oct) on the proportion of dominant females that produce litters per year [31] and other research has found that prior reproductive effort influences reproductive output in North American beavers (Ruusila et al. 2000). Combining these factors, to test whether *TQ_4_* affects dominant female reproductive success, we constructed a global model that initially included minimum maternal age (*mmage*) and its quadratic term (*mmage^2^*), along with rainfall in the previous autumn (*rain*), reproductive success in previous year (a binary variable: *RPY*) and *TQ_4_*. We included two-way interactions of *mmage* with *rain*, *RPY* and *TQ_4_*. To investigate whether differential a mortality rate (SH) accounted for the decline in reproductive output with age we observed at the population level (see results) , we added the maximum age at which a dominant female was last detected (*maxage*), and a *maxage* × *mmage* interaction. Variation in group sizes could modify the effects of *TQ_4_*. However, group sizes were highly dependent on *RPY* (see below). Trapping effort (*TE*) was also included, as this could influence ability to detect offspring. *Mmage*, *rain* and *TE* were all centred at zero mean and unit variance (*mmage* mean = 6.44, SD = 2.92; *rain* mean = 324.6, SD = 113.7; *TE* mean = 22.9, SD = 12.5). *TQ_4_* was centred at median = 3 and SD = 1.17. We then reduced this global model by eliminating all possible combinations of predictors, except trapping effort.

For the model of litter size, because mothers >8 years produced relatively few kits (n kits = 13/106), we combined minimum maternal ages ≥9. *Mmage39* and *mmage39^2^* were centred as described above (mean = 5.70, SD = 1.91). We included two-way interactions of *mmage39* with *rain* and *TQ_4_*. We then reduced this global model by eliminating all possible combinations of predictors, except trapping effort.

Offspring quality and mother age

From initial data exploration, juvenile beaver *BW* and *BL* increased with age. We therefore included an age term (*seasonage*), to identify whether an individual was first measured as a kit in summer (Jul-Aug) or autumn (Sept-Nov) or as a yearling in the following spring (Mar-May), summer (Jun-Aug) or autumn. *Seasonage*, along with *lnBL* (models of *BW* only), were included as control variables in all candidate models.

Litter size (*LS*) may influence offspring quality. Due to sample size constraints, *LS*= 4 was combined with *LS*= 3. Again minimum maternal ages (*mmage*) ≥9 were combined. Since we expected a directional relationship with both *LS* and *mmage*, we nevertheless treated both variables as continuous. We included a quadratic term for *mmage* in the full model, because effects on offspring body condition might correspond with the probability of reproductive senescence.

In addition, the global model included: *TQ_4_* and a binary rainfall variable (*rainbin*) describing whether rain from Apr – Sept (median 570mm 1998-2011) in the birth year (inversely related to body weight in beaver kits in the study area, [31]) was <570mm (-1) or ≥570mm (+1). *TQ_4_* was centred at median (3) and unit variance (0.984) while litter size was centred as before.

Effects or reproductive success in the previous year (RPY) on group sizes.

Initially data exploration indicated that *RPY* was highly correlated with group size (excluding young of the year) (Kendall correlation, z = 3.87, df = 168, p = 0.0001). To explore this further, we used a GLMM with a Poisson error structure to test the effect of *RPY* on group size. We included a random intercept for territory (i.e. family) and compared the global model with a null model. The global model was most supported, with the null model exhibiting a ΔAICc of +5.93. The estimate from the global model of *RPY* on group size indicated a significant positive effect (estimate = 0.254, 95% CIs 0.081 – 0.427, z = 2.88, r^2^c = 0.198).

**References, additional to main text:**

Freckleton RP. On the misuse of residuals in ecology: Regression of residuals vs. multiple regression. *Journal of Animal Ecology* 2002; 71:542-545.

Haarberg O, Rosell F. Selective foraging on woody plant species by the Eurasian beaver (*Castor fiber*) in Telemark, Norway. *Journal of Zoology* 2006; 270:201-208.

Parker H, Haugen A, Kristensen Ø, Myrum E, Kolsing R, Rosell, F. Landscape use and economic value of Eurasian beaver (*Castor fiber*) on a large forest in southeast Norway. *In: Proceedings 1st European-American Beaver Congress*. Kazan. 2001.

Rosell F, Thomsen L. Sexual Dimorphism in Territorial Scent Marking by Adult Eurasian Beavers (*Castor fiber*). *Journal of Chemical Ecology* 2006; 32:1301-1315.

Rosell F, Bergan F, Parker H. Scent-marking in the Eurasian beaver (*Castor fiber*) as a means of territory defense. *Journal of Chemical Ecology* 1998; 24:207-219.

Ruusila V, Ermala A, Heikki H. Costs of reproduction in introduced female Canadian beavers (*Castor canadensis*). *Journal of Zoology* 2000; 252:79-82.

Schielzeth H, Forstmeier W. Conclusions beyond support: overconfident estimates in mixed models. *Behavioral Ecology* 2009; 20:416-420.
