# Supplementary material for: Age-related changes in somatic condition and reproduction in the Eurasian beaver: Resource history influences onset of reproductive senescence
Source: PLoS One. 2017 Dec 5;12(12):e0187484. doi: 10.1371/journal.pone.0187484 (PMC5716577; doi:10.1371/journal.pone.0187484)
Supplement: S2 File — (DOCX) [file pone.0187484.s002.docx]

## S2 File. Comparison with other studies on beaver

To date, substantiating senescence in the North American beaver has been limited by small sample sizes for older individuals; however, inference supports a slight decline in the proportion of pregnant females in older cohorts (Henry and Bookhout 1969; Payne 1984). There are several potential reasons why the senescence signal proved much stronger in our study of Eurasian beavers. Firstly, previous studies have been cross-sectional, and thus unable to assess whether differential mortality (SH, [16]) masked a senescent decline in reproductive success; in contrast our longitudinal study allowed examination of individual life-histories. Secondly, the North American beaver studies by Henry and Bookhout (1969) and Payne (1984) examined pregnancy rates across ‘*all*’ females of reproductive age – not just territory holders. This would include non-breeding subordinate animals, reducing the apparent pregnancy rate in younger animals, but not older animals, because older survivors are more likely to hold dominant positions [27]. Thirdly, both North American studies used a combination of *corpora lutea*, placental scars and embryos to assess pregnancy, whereas we used the number of offspring surviving until emergence at least two-months old. It is feasible that older female Eurasian beavers produce as many embryos as younger animals, but experience greater losses of kits prior to emergence.

**References, additional to main text:**

Henry DB, Bookhout TA. Productivity of Beavers in Northeastern Ohio. *Journal of Wildlife Management* 1969 33:927-932.

Payne NF Reproductive Rates of Beaver in Newfoundland. *Journal of Wildlife Management* 1984; 48:912-917.
