# Supplementary material for: Age-related changes in somatic condition and reproduction in the Eurasian beaver: Resource history influences onset of reproductive senescence
Source: PLoS One. 2017 Dec 5;12(12):e0187484. doi: 10.1371/journal.pone.0187484 (PMC5716577; doi:10.1371/journal.pone.0187484)
Supplement: S1 Fig — Surface plot of the interaction between maternal age, territory quality and probability of reproduction. (DOCX) [file pone.0187484.s006.docx]

## S1 Fig. Relationship between reproduction, age and territory quality


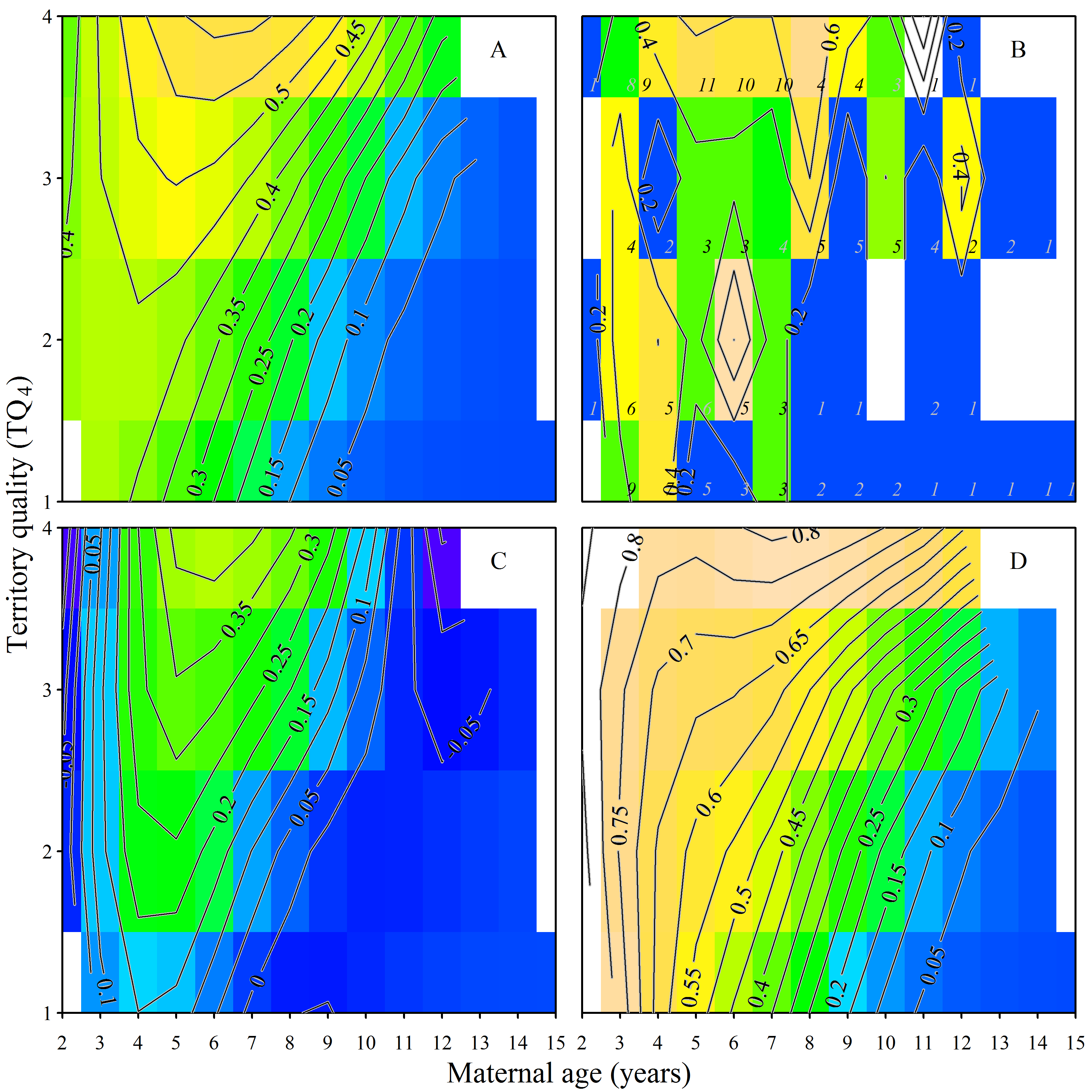


Response surface of the probability of reproduction (contours and shading) with mother minimum age (*mmage*) and territory quality (*TQ_4_*). Shading graduates from blue (low), through green, yellow and pink to white (high), while white cells without contours represent *TQ_4_* × *mmage* combinations where no data were available. Plot a) is the response surface predicted from the averaged top set of GLMM models; b) summarizes the raw data with sample sizes given at the bottom right of each cell; c) is the lower and d) is the upper 95% prediction intervals around a).
