# Supplementary material for: Age-related changes in somatic condition and reproduction in the Eurasian beaver: Resource history influences onset of reproductive senescence
Source: PLoS One. 2017 Dec 5;12(12):e0187484. doi: 10.1371/journal.pone.0187484 (PMC5716577; doi:10.1371/journal.pone.0187484)
Supplement: S2 Fig — (DOCX) [file pone.0187484.s007.docx]

## S2 Fig. Individual quality and territory quality

Campbell [38] found that longer beavers were more likely to achieve dominant breeding positions in this population, though without reference to territory quality. That is, ‘better’ individuals typically exhibit greater reproductive output, but might also occupy higher quality territories. As a consequence, smaller beavers would be unlikely to gain and hold dominant breeding positions, and so the largeness of the beaver precedes the quality of the territory held.

We examined body length and condition of 41 individuals (18 males and 23 females) for which we had data from the first trapping occasion after they gained a dominant breeding position. Of these, the maximum time each could have held the dominant position (based on the disappearance of previous incumbents) was <1 year for 26 individuals, one year for five individuals and 2-6 year for the remaining 10 individuals (mean = 1.1 years ± 1.8 SD). Due to restricted sample size, instead of modelling body condition, as we did for the somatic senescence model (see S1 Appendix), we calculated a body condition index (*BCI*), based on observed weight minus weight predicted from length, time of year and age. *BCI* was derived from the model of somatic senescence, described in the main text, as observed *lnBW* – predicted *lnBW* (kg). Accordingly, we constructed a LMM using function *lmer* in the *R* package *lme4* [42], in R 2.14 [41] with body length (*lnBL*, cm) and *BCI* as predictors, and territory quality (*TQ_4_*) as the response variable. Random intercepts were specified for year (random intercept model, Schielzeth and Forstmeier 2009). A Gaussian distribution was specified with an identity link function. *BCI* and *lnBL* were centred at zero mean and unit variance, where the mean and SD was: *BCI* = 0.0046 ± 0.090 and *lnBL* = 4.37 ± 0.044.

We thus constructed the global model:

TQ_4_ = BCI + lnBL

We produced a candidate model set of four models by reducing this global model to all possible combinations of fixed effects, including a null model.

Results and discussion

Neither *BCI* (estimate = -0.151, 95% CIs = -0.442 to 0.140) nor *lnBL* (estimate = 0.051, 95% CIs = -0.240 to 0.342) predicted the quality of the territory held, averaged over the three most supported models, which ranked ∆AICc<4; and the null model ranked top (Akaike weight = 0.505, Table C1). The global model was outside this top set (∆AICc = 5.24).

Fourteen individuals in this dataset (seven female and seven male) were of know age at the time they acquired breeding positions. The mean age at which they were known to have attained their breeding status was 5.1 years ± 1.8 SD (female 5.0 ± 2.0 years; male 5.1 ± 1.7 years, range 3 – 8 for both sexes).

We found no evidence for better quality females occupying, on average, better quality territories. Larger and heavier (for their length) individuals (of both sexes) attained territories similar in quality to smaller and lighter animals.

Vacancies in territory ownership, in this densely populated study area, were sufficiently rare (mean = 12% of female breeding positions becoming available per year), and territory fidelity sufficiently high (of 57 breeding females, only two changed territory) that, tactically, individuals prospecting to acquire a territory would most likely take the first to become available (i.e., their first opportunity to pair and breed).

##### Table S3: Model selection table examining the effects of individual quality (body condition index, BCI, and ln body length, lnBL) on territory quality (TQ_4_).

| Intercept | BCI | lnBL | BCI × lnBL | df | logLik | AICc | ∆AICc | Akaike weight |
| --- | --- | --- | --- | --- | --- | --- | --- | --- |
| 2.512 |  |  |  | 3 | -55.649 | 117.9 | 0.00 | 0.505 |
| 2.593 | -0.1508 |  |  | 4 | -55.216 | 119.5 | 1.60 | 0.227 |
| 2.512 |  | 0.05089 |  | 4 | -55.590 | 120.3 | 2.35 | 0.156 |
| 2.603 | -0.1435 | 0.06583 |  | 5 | -55.134 | 122.0 | 4.04 | 0.067 |
| 2.562 | -0.0637 | 0.04923 | 0.217 | 6 | -54.187 | 122.8 | 4.90 | 0.044 |

***References, additional to main text***

Schielzeth H, Forstmeier W. Conclusions beyond support: overconfident estimates in mixed models. *Behavioral Ecology* 2009; 20:416-420.
